# Mitochondrial genome complexity in Erodium stephanianum (Geraniaceae): nanopore sequencing reveals chloroplast gene transfer and DNA rearrangements

**DOI:** 10.3389/fgene.2025.1641368

**Published:** 2025-07-15

**Authors:** Xinchen Xu, Qingfei Meng, Na Li, Zichuan Zou, Haonan Yang, Ang Li, Fusheng Ge, Jian Meng, Zixue Ding

**Affiliations:** ^1^ Xuzhou Central Hospital, Xuzhou Clinical School of Xuzhou Medical University, Xuzhou, Jiangsu, China; ^2^ Southeast University affiliated Xuzhou Central Hospital, Xuzhou, Jiangsu, China; ^3^ Jiangsu Key Laboratory of New Drug Research and Clinical Pharmacy, Xuzhou Medical University, Xuzhou, Jiangsu, China

**Keywords:** Erodium stephanianum Willd., mitochondrial genome (mitogenome), repeated sequences (RS), phylogenetic relationship analysis, RNA editing

## Abstract

The mitochondrial genome of Erodium stephanianum (Geraniaceae) exhibits remarkable complexity revealed through nanopore sequencing, which has unveiled both chloroplast-to-mitochondrion gene transfer and extensive DNA rearrangements. We collected leaf samples from E. stephanianum in June 2023, subsequently extracting total DNA and sequencing the mitogenome using both Oxford Nanopore and Illumina technologies. The assembly yielded a circular mitochondrial genome of 365,414 base pairs, encompassing 28 unique protein-coding genes, 18 tRNA genes, and 3 rRNA genes. Notably, 55 fragments, totaling 58,305 base pairs, showcased sequence homology between the chloroplast and mitochondrion, indicating substantial gene transfer with implications for evolutionary adaptation. Furthermore, codon usage analysis revealed preferential codon utilization, while microsatellite and repeat sequence analyses identified numerous SSRs and tandem repeats within the mitogenome. Phylogenetic analysis positioned E. stephanianum within the Geraniales order, closely clustering with Geranium maderense. This study highlights the dynamic evolution of mitochondrial genomes in E. stephanianum, emphasizing the significance of interorganellar gene transfer and genome rearrangement.

## 1 Introduction

Erodium stephanianum (Erodium stephanianum Willd. Sp. Pl. 1800; E. stephanianum).

The mitochondrial genome is a critical component of the cellular energy production system in eukaryotic organisms (Carelli and Chan, 2014), and its complexity has become a focal point of genomic research. In plants, mitochondrial genomes often exhibit considerable variation in structure and gene content (Choi et al., 2021; Abu-Elmakarem et al., 2024; Lareau et al., 2023), influenced by evolutionary processes such as gene transfer and rearrangements (Li et al., 2022a; Robison et al., 2022; Xie et al., 2024) Erodium stephanianum (Geraniaceae) serves as an intriguing model for investigating these phenomena, particularly in relation to chloroplast-to-mitochondrion gene transfers, which underscore the evolutionary interplay between these organelles. E. stephanianum contain certain bioactive compounds that could be beneficial for oral health, such as anti-inflammatory, antibacterial, or antioxidant properties. E. stephanianum also be used as traditional remedies in some regions (Zhang et al., 1995; Yin et al., 2010).

Recent advancements in sequencing technologies, notably nanopore sequencing (Karin et al., 2023; Akamatsu et al., 2024), have facilitated the comprehensive analysis of mitochondrial genomes, revealing insights into their genomic architecture and functional capacities. The mitochondrial genome of E. stephanianum presents an opportunity to unravel the complexities associated with interorganellar gene transfer and extensive DNA rearrangements. Previous studies have documented the presence of chloroplast-derived sequences within mitochondrial genomes (Xie et al., 2024; Cui et al., 2021), an observation that raises questions about the mechanisms and implications of such genetic exchanges.

In this study, we present a detailed investigation of the mitochondrial genome of E. stephanianum, leveraging both nanopore and Illumina sequencing platforms to achieve high-resolution genomic data. Our findings reveal a circular mitochondrial genome of 365,414 base pairs, encompassing 28 unique protein-coding genes, in addition to tRNA and rRNA genes. We also identify significant homologous fragments between the chloroplast and mitochondrial genomes, highlighting the extent of gene transfers. Furthermore, we explore codon usage patterns and repetitive sequences within the mitogenome, echoing broader trends observed in plant mitochondrial evolution.

By elucidating the complexities of the mitochondrial genome in E. stephanianum, this study enhances our understanding of interorganelle interactions and their evolutionary significance. The results provide a foundation for future research into the dynamics of mitochondrial genome evolution and its implications for plant adaptability.

## 2 Materials and methods

### 2.1 Materials and sequencing

In June 2023, live E. stephanianum leaves were collected from Moyun Mountain, Jinan City, Shandong Province (36°20′31.0308″N, 117°54′43.4772″E) (Gao et al., 2024). E. stephanianum was not an endangered or protected species and specific permission for the collection of E. stephanianum was not required.

All samples were thoroughly rinsed, cleaned using DEPC water, and subsequently stored at −80°C. Total DNA was extracted using TIANamp Genomic DNA Kit (Tiangen, Beijing, China). To obtain comprehensive data, we sequenced the mitogenome of E. stephanianum on both Nanopore GridION sequencing platform (Pugh, 2023) (Oxford Nanopore Technology, Oxford Science Park) and Illumina Novaseq 6,000 platform (Illumina, San Diego, United States), which enabling the construction of libraries and the generation of raw data (Nanopore raw data: 30.86 Gb, Illumina raw data: 24 Gb). The data reported in this paper have been deposited in the GenBank of NCBI (Sayers et al., 2025), under accession number PV575339 that were publicly accessible at https://www.ncbi.nlm.nih.gov/genbank/.

### 2.2 Assembly and annotation of organelle genomes

The E. stephanianum mitogenome was assembled using a comprehensive approach combining Illumina and Nanopore sequencing technologies. Initially, we employed Flye (Freire et al., 2022) software to conduct *de novo* assembly of long reads derived from E. stephanianum obtained through Oxford Nanopore sequencing. Subsequently, the BLASTn (Chen et al., 2015) was utilized to identify the draft mitogenome of E. stephanianum by comparing the assembled contigs. To facilitate this process, we created a database for the assembled sequences using makeblastdb and chose conserved mitochondrial genes from *Arabidopsis thaliana* (L.) Heynh. as our query sequence to pinpoint contigs that contain these conserved mitochondrial genes. The commonly parameters used for this assembly included “-evalue 1e-5 -outfmt 6 -max_hsps 10 -word_size 7 -task blastn-short”. Additionally, we conducted a hybrid assembly using Unicycler, intergrating both Illumina short reads and Nanopore long reads (Schafer et al., 2024). For the annotation of protein-coding genes (PCGs) in the mitogenome, we selected A. thaliana (NC_037304) and Liriodendron tulipifera (NC_021152.1) as reference genomes, using Geseq for the annotation process (Tillich et al., 2017). Annotation of tRNA and rRNA within the mitogenome was accomplished using tRNAscan-SE (Chan et al., 2021) and BLASTn (Chen et al., 2015), respectively. Manual correction of annotation errors in the mitogenome was performed using Apollo (Dunn et al., 2019).

### 2.3 Analysis of codon usage and repeated sequences

Protein-coding gene (PCG) sequences were extracted from the genome using Phylosuite (Xiang et al., 2023), and the codon usage in mitochondrial PCGs was analyzed using Mega 7.0 (Kumar et al., 2016) and relative synonymous codon usage (RSCU) values were also calculated. To identify repeated sequences, including simple sequence repeats (SSRs), tandem repeats, and interspersed repeats, MISA (Beier et al., 2017), TRF (Benson, 1999), and REPuter (Kurtz et al., 2001) were employed. The results were visualized using the RCircos (Zhang et al., 2013) package.

### 2.4 Prediction of RNA editing sites

Deepred-mt (Edera et al., 2021), a tool based on the convolutional neural network (CNN) model, was utilized for predicting C to U RNA editing sites. Mitochondrial protein-coding genes were extracted for prediction analysis, and only results with probability values exceeding 0.9 were selected for further consideration.

### 2.5 Chloroplast to mitochondrion DNA transformation

The chloroplast genome was assembled and annotated using GetOrganelle (Jin et al., 2020), and CPGAVAS2 (Shi et al., 2019), respectively. The BLASTn (Chen et al., 2015) program was utilized to compare two organelle genomes of E. stephanianum. In this process, the mitogenome was established as the database with makeblastdb, and the chloroplast genome was employed as the query sequence. All results were visualized using the RCircos (Zhang et al., 2013) package.

### 2.6 Phylogenetic inference

Related species of E. stephanianum were selected based on their genetic relationship, and their complete mitogenome sequences were downloaded from NCBI (https://www.ncbi.nlm.nih.gov) (Supplementary Table S3). PhyloSuite was utilized to extract shared mitochondrial genes across these species. Multiple sequences alignment was carried out using MAFFT (Katoh et al., 2019) with a bootstrap value of 1,000. IQ-TREE (Minh et al., 2020) was used for phylogenetic analysis. The resulting phylogenetic analysis was visualized using iTOL (Letunic and Bork, 2021).

### 2.7 Synteny analysis

Using the BLASTn, we identified conserved homologous sequences, which are referred to as co-linear blocks, with commonly parameters “-value 1e-5, -word_size 9, -gapopen 5, -gapextend 2, -reward 2, -penalty -3”. As a result, only co-linear blocks longer than 500 bp were considered. Based on sequence similarity. The mitochondrial genome of E. stephanianum was compared with multiple synteny regions from closely related species using MCscanX (Wang et al., 2024).

## 3 Results

### 3.1 Characteristics of the mitochondrial genomes of E. stephanianum

**FIGURE 1:**
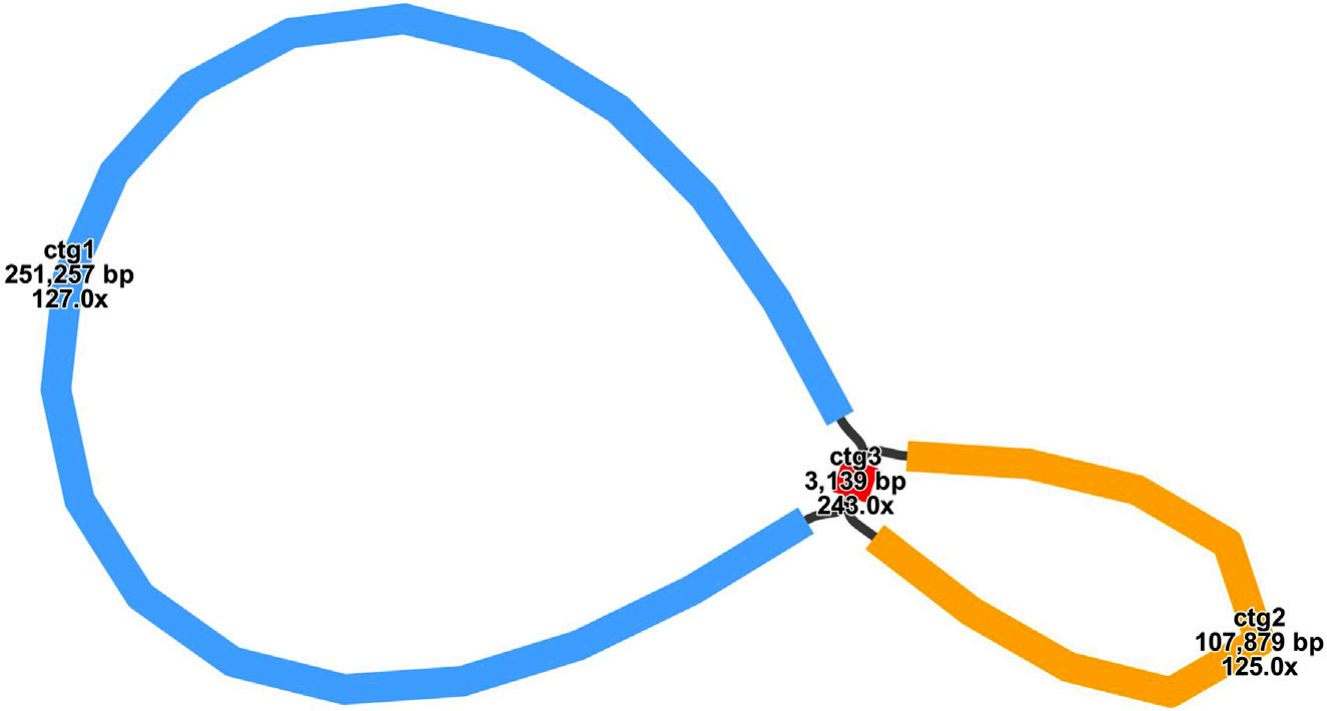
The assembly graph of the mitochondrial genome of E. stephanianum. Each colored segment is labeled with its size and named contig1-3 by rank of size. Only segment contig3 representation is inferred as repeats. All segment adjacencies are supported by the long reads, indicating a complex branching genomic structure.

The mitochondrial genome of E. stephanianum exhibits a circular structure. We utilized Bandage software to visualize the draft mitochondrial genome assembled from long-read data. The final results are illustrated in Figure 1, which includes three nodes (Alternative conformations are illustrated in Supplementary Figures S1, S2). The assembly graph of the mitochondrial genome of E. stephanianum includes three distinct contigs/nodes (ctg). The ctg1 has a length of 251,257 base pairs; the ctg2 measures 107,879 base pairs in length; the ctg3 is significantly shorter, at 3,139 base pairs. Detailed information can be found in Supplementary Table S1.

**FIGURE 2:**
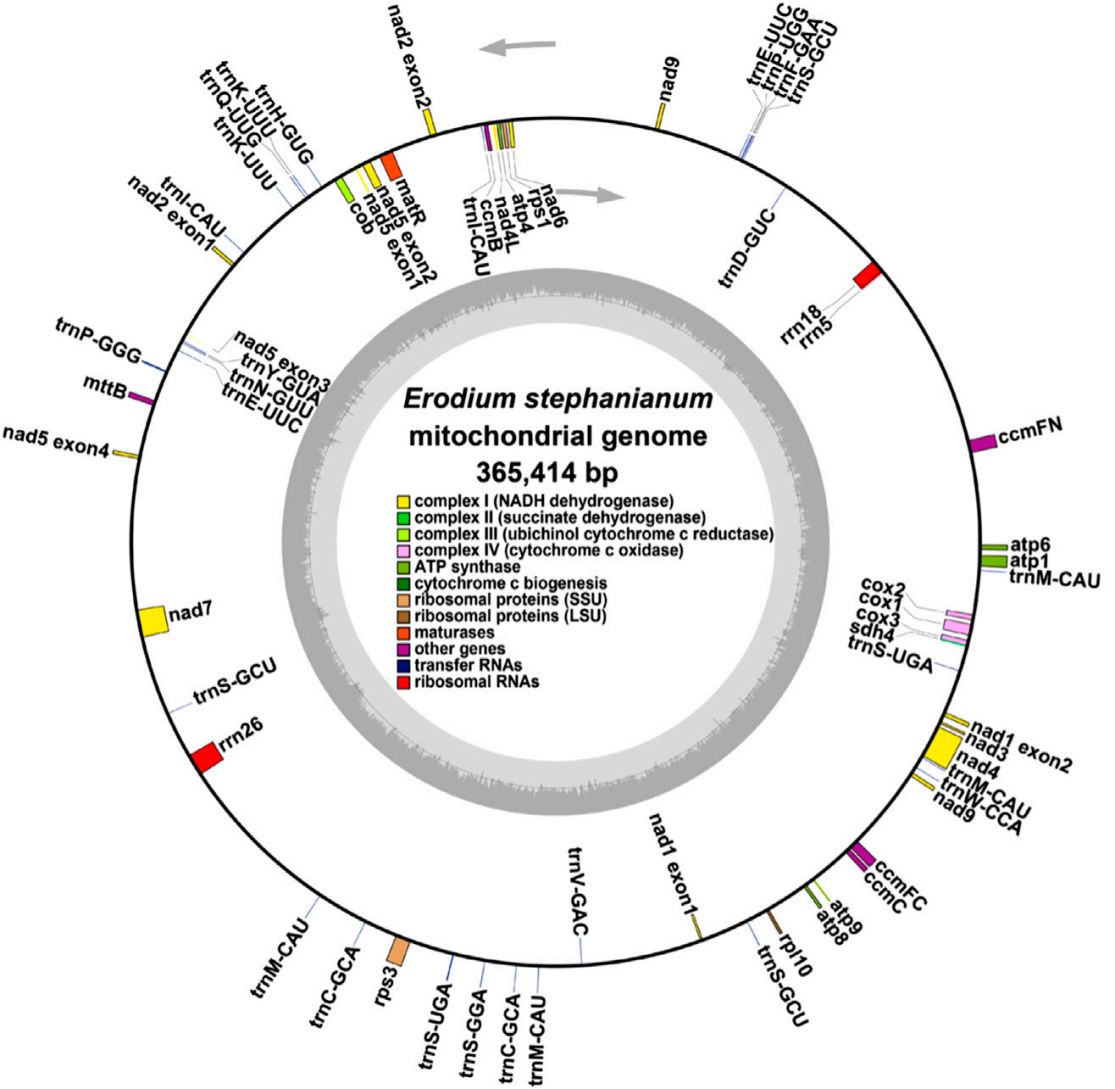
E. stephanianum mitogenome gene map. Genes shown on the outside and inside of the circle are transcribed clockwise and counterclockwise, respectively.

The mitochondrial genome of E. stephanianum is primarily structured as a single circular molecule. After eliminating duplicate regions using Nanopore sequencing data, we identified a predominant circular contig with a total length of 365,414 bp and a GC content of 42.72% (Supplementary Table S2). Annotation of the mitochondrial genome yielded a total of 28 unique protein-coding genes, which include 24 core mitochondrial genes and four non-core genes, as well as 18 tRNA genes (of which 7 are multicopy) and 3 rRNA genes. The core genes comprise 5 ATP synthase genes (atp1, atp4, atp6, atp8, and atp9), 9 NADH dehydrogenase genes (nad1, nad2, nad3, nad4, nad4L, nad5, nad6, nad7, and nad9), 4 cytochrome c biogenesis genes (ccmB, ccmC, ccmFC, and ccmFN), 3 cytochrome c oxidase genes (cox1, cox2, and cox3), one membrane transport protein gene (mttB), one maturation enzyme gene (matR), and one coburol-ferredoxin reductase gene (cob). The non-core genes include one large ribosomal subunit gene (rpl10), two small ribosomal subunit genes (rps1 and rps3), and one succinate dehydrogenase gene (sdh4) (Figure 2; Table 1).

**TABLE 1:** Mitochondrial-encoded genes of E. stephanianum.

Group of genes	Name of genes
ATP synthase	atp1,atp4,atp6,atp8,atp9 (×2)
NADH dehydrogenase	ad1,nad2,nad3,nad4,nad4L,nad5,nad6, nad7,nad9
Cytochrome b	cob
Cytochrome c biogenesis	ccmB, ccmC,ccmFC, ccmFN
Cytochrome c oxidase	cox1,cox2,cox3
Maturases	matR
Protein transport subunit	mttB
Ribosomal protein large subunit	rpl10
Ribosomal protein small subunit	rps1,rps3
Succinate dehydrogenase	sdh4
Ribosome RNA	rrn5,rrn18,rrn26
Transfer RNA	trnC-GCA (×2),trnD-GUC,trnE-UUC(×2),trnF- GAA,trnH-GUG,trnI-CAU(×2),trnK-UUU (×2),trnM-CAU(×4),trnN-GUU,trnP-GGG, trnP-UGG,trnQ-UUG,trnS-GCU(×3),trnS- GGA,trnS-UGA(×2),trnV-GAC,trnW-CCA, trnY-GUA

^a^ The numbers in parentheses represent the copy number of the gene, (×2) indicates that there are two copies.

**FIGURE 3:**
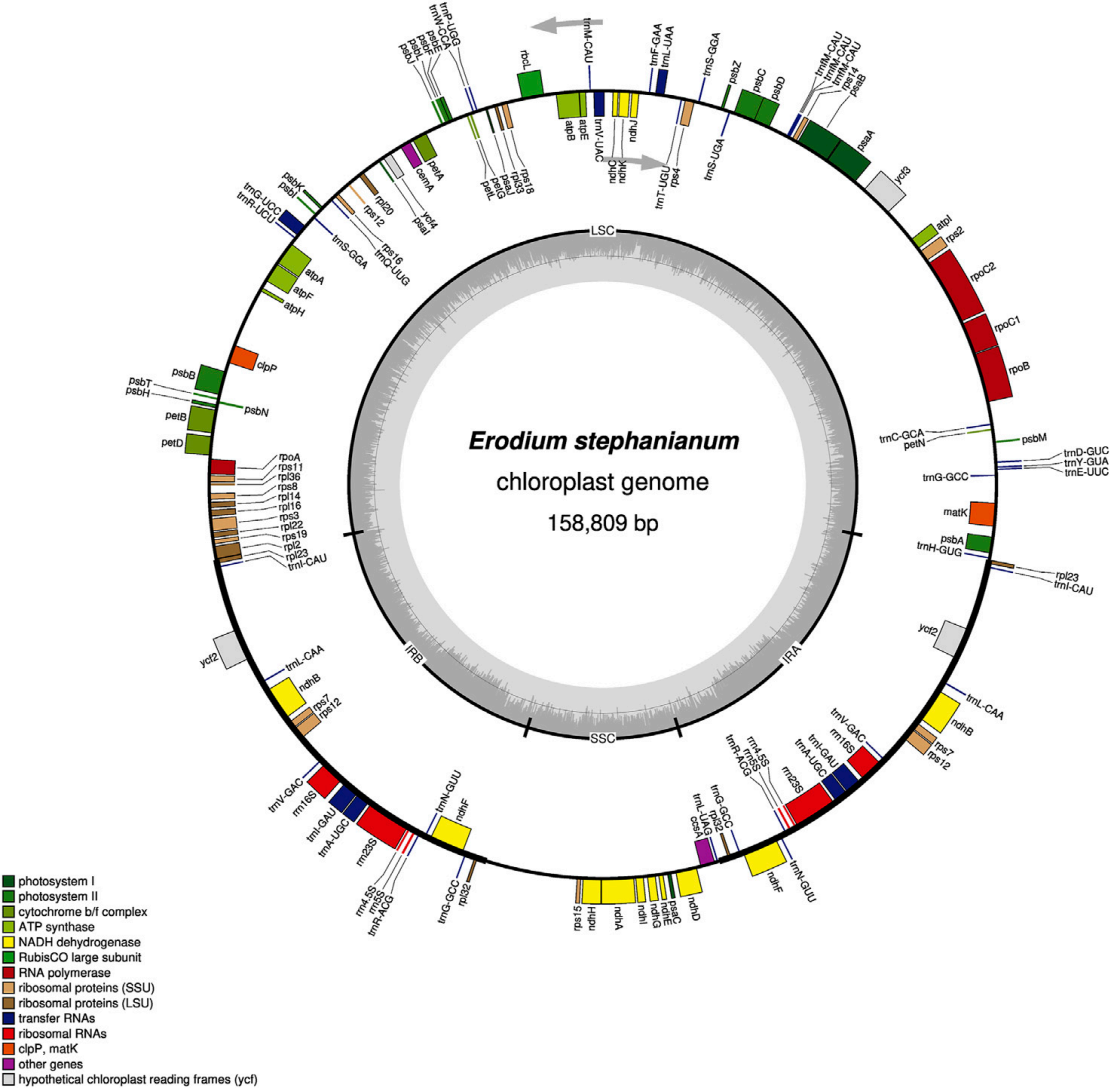
The chloroplast genome map of E. stephanianum. Genes on the inside of the circle were transcribed in a clockwise direction and genes on the outside of the circle were transcribed in a counterclockwise direction.

In our previous study, we present the first annotated chloroplast genome of E. stephanianum, describing its structure. The genome has a total length of 158,809  bp and contains 76 annotated protein-coding genes. Phylogenetic analysis confirms that E. stephanianum belongs to the Erodium genus in the family Geraniaceae (Gao et al., 2024) (Figure 3). Interestingly, several plastid genes were also annotated in the mtDNA, albeit mostly as fragments. These include rps3, atp1, trnC-GCA, et al. This finding suggests a notable sequence migration was observed between the chloroplast DNA (cpDNA) and mtDNA of E. stephanianum, which was accompanied by gene transfer, which will be discussed in detail below.

### 3.2 PCGs codon usage analysis

**FIGURE 4:**
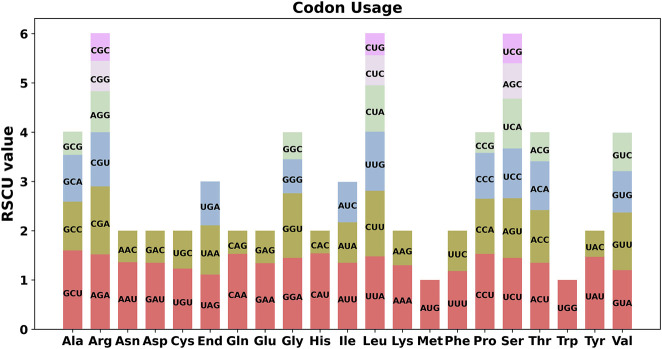
E. stephanianum mitogenome relative synonymous codon usage (RSCU). Codon families are shown on the x-axis. RSCU values are the number of times a particular codon is observed relative to the number of times that codon would be expected for a uniform synonymous codon usage.

An analysis of codon preference among the 28 unique protein-coding genes (PCGs) of E. stephanianum mitochondria was conducted, with the usage of various codons for each amino acid summarized in Supplementary Table S3. Codons with a relative synonymous codon usage (RSCU) greater than one are considered to be favored by their respective amino acids. As illustrated in Figure 4, with the exception of the start codon AUG and the tryptophan codon (UGG), which both have an RSCU value of 1, there is a general preference for codon usage among the mitochondrial PCGs. For instance, alanine (Ala) exhibits a strong preference for the codon GCU, achieving the highest RSCU value of 1.6 among the mitochondrial PCGs, followed by histidine (His), which favors the codon CAU with an RSCU value of 1.54. Notably, the highest RSCU values for phenylalanine (Phe) and stop codons are both below 1.2, indicating a lack of strong codon usage preference.

### 3.3 E. stephanianum mitogenome repeats analysis

**FIGURE 5:**
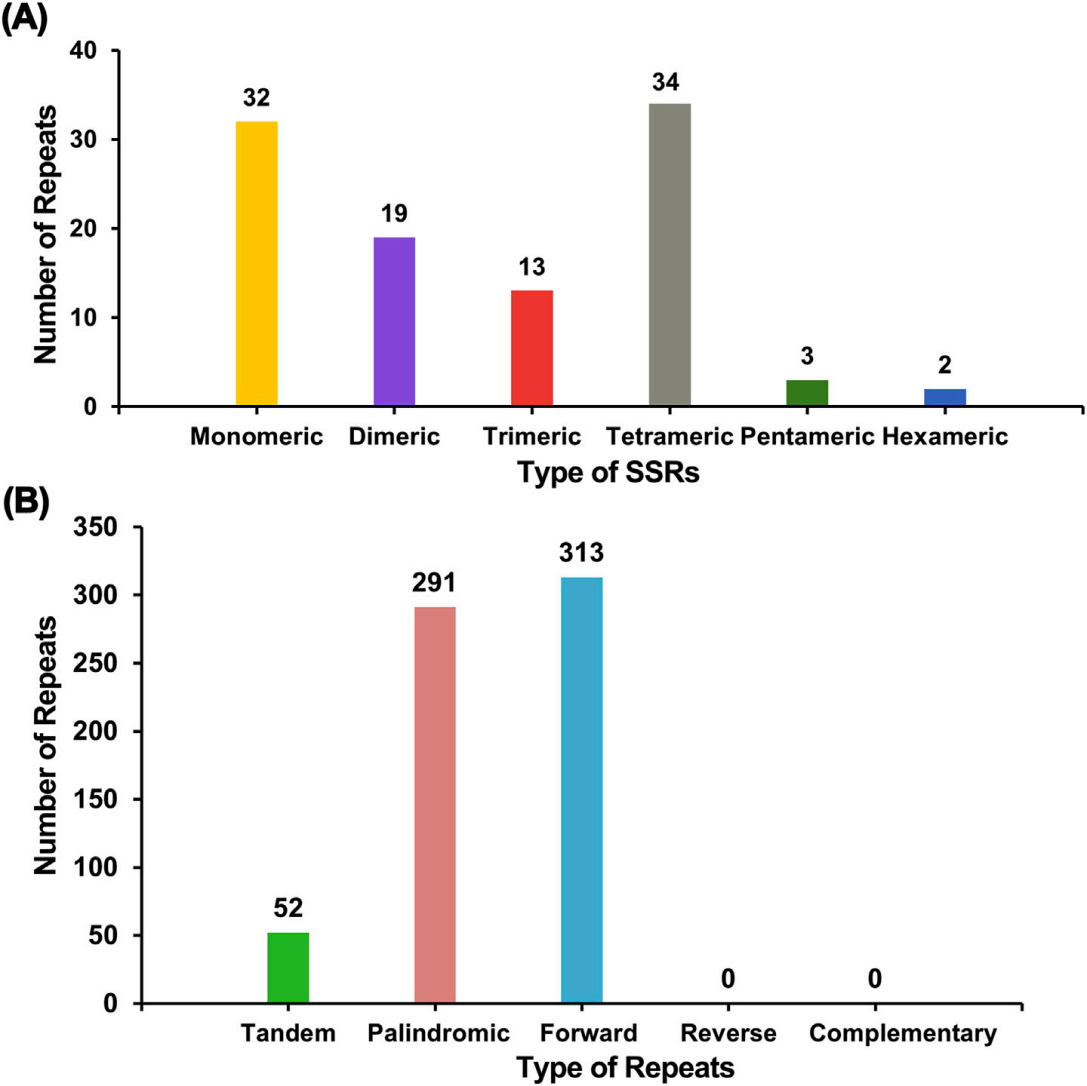
The distribution of repeats in the mitogenome of E. stephanianum. **(A)** The x-axis denotes the types of simple sequence repeats (SSRs), while the y-axis represents the quantity of repeat fragments. The yellow legend indicates monomeric SSRs, the purple legend indicates dimeric SSRs, the red legend indicates trimeric SSRs, the gray legend indicates tetrameric SSRs, the green legend indicates pentameric SSRs, and the blue legend indicates hexameric SSRs. **(B)** The x-axis signifies the types of repeat sequences, and the y-axis displays the quantity of repeat fragments. The green legend represents tandem repeats, the red legend indicates palindromic repeats, and the blue legend signifies forward repeats.

Microsatellites, also known as simple repeat sequences (SSRs), were analyzed to determine the presence of repeat sequences in the mitogenome. An analysis of repetitive sequences in the E. stephanianum mitochondrial genome revealed a total of 103 simple sequence repeats (SSRs), with monomeric and dimeric forms accounting for 49.51% of the total SSRs (Supplementary Table S4). Thymidine (T) monomeric repeat sequences comprised 50.00% (16 out of 32) of the monomeric SSRs. Tandem repeats, also known as satellite DNA, are characterized by core repeating units of approximately 7–200 bases that are repeated in succession. These sequences are widely distributed in the genomes of eukaryotes and prokaryotes. Within the E. stephanianum mitochondrial genome, 52 tandem repeat sequences were identified, with a similarity greater than 76% and lengths ranging from 9 to 43 bp. Additionally, scattered repeats within the Es mitochondrial genome were detected, yielding a total of 604 repeat sequences with lengths of 30 bp or greater. This included 291 palindromic repeats, 313 forward repeats, and no instances of reverse repeats or complementary repeats. The longest palindromic repeat measured 704 bp, while the longest forward repeat extended to 3,139 bp. A bar char in Figure 5 illustrates the different types of repeat sequences.

### 3.4 Chloroplast to mitochondrion DNA transformation

**FIGURE 6:**
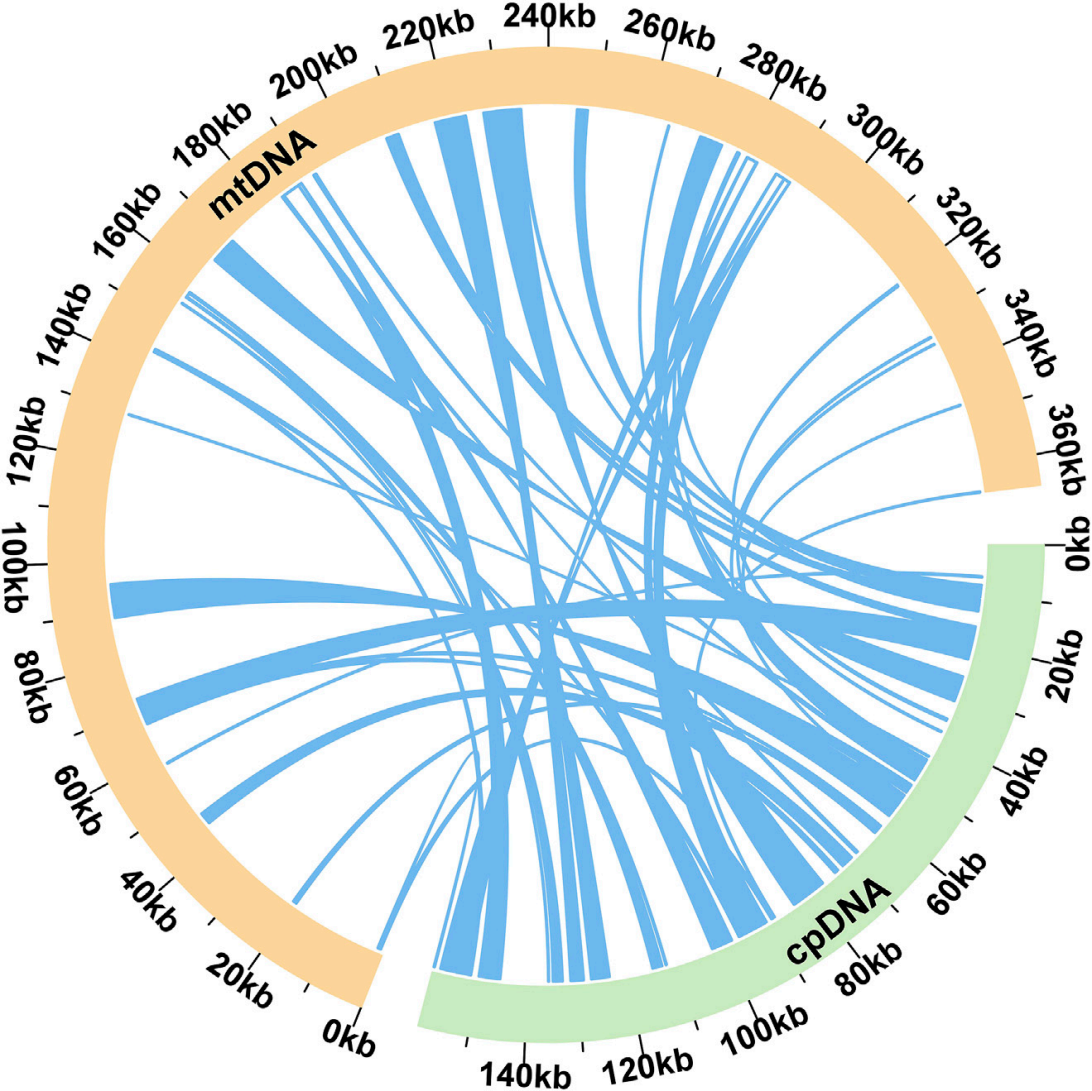
Schematic representation of homologous sequences between chloroplast genome and mitogenomes in E. stephanianum. The yellow arcs represent mitogenomes, the green arcs represent chloroplast genomes, and the blue lines between arcs correspond to homologous genome segments (co-linear blocks).

We observed significant sequence transfers from the chloroplast genome to the mitogenome in E. stephanianum. Based on sequence similarity analysis, a total of 55 segments were identified as homologous fragments between the mitochondrial and chloroplast genomes, with an aggregate length of 58,305 bp, which accounts for 15.96% of the total mitochondrial genome length (Figure 6). The longest segment, MTPT22, measures 5,586 bp. Annotation of these homologous sequences revealed the presence of 33 complete genes within the 55 homologous fragments, including 28 protein-coding genes (atpB, atpE, atpF, atpH, atpI, ccsA, ndhA, ndhI, petA, petB, petD, petG, petL, petN, psaJ, psbB, psbF, psbJ, psbL, psbN, psbT, rpl33, rpoA, rpoC2, rps18, rps2, rps7, ycf2) and five tRNA genes (trnC-GCA, trnD-GUC, trnM-CAU, trnN-GUU, trnW-CCA) (Supplementary Table S5).

### 3.5 Synteny analysis and phylogenetic inference

**FIGURE 7:**
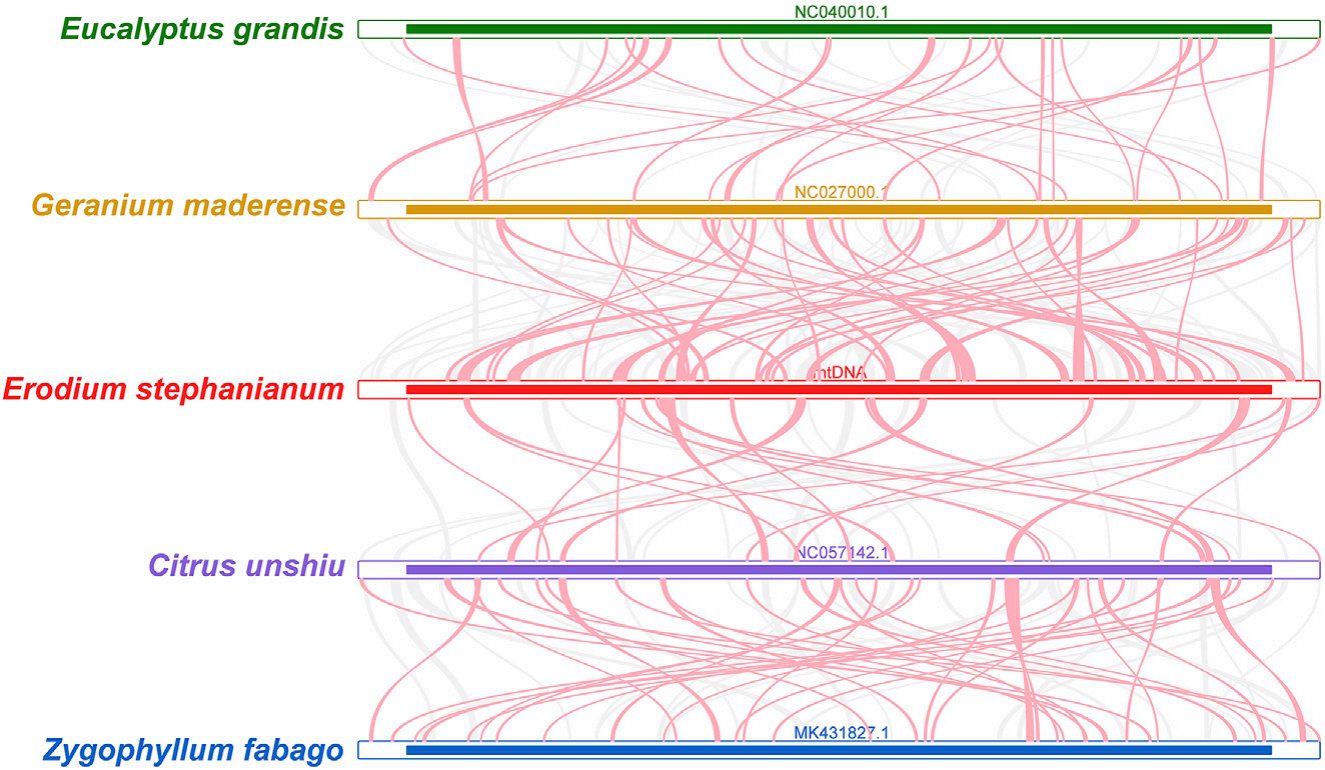
Mitogenome synteny. Bars indicated the mitogenomes, and the ribbons showed the homologous sequences between the adjacent species. The red areas indicate where the inversion occurred, the gray areas indicate regions of good homology. Common blocks less than 0.5 kb in length are not retained, and regions that fail to have a common block indicate that they are unique to the species.

To investigate the synteny relationship between E. stephanianum and closely related species, we utilized MCscanX to generate multiple synteny plots based on the sequence similarity. Figure 7 illustrates that the co-linear blocks exhibit varying arrangements across individual mitochondrial genomes, the red arched regions represent inverted sequences, while the gray areas indicate regions with high sequence homology. Although a substantial number of blocks were detected between E. stephanianum and Geranium maderense and Citrus unshiu, these co-linear blocks appeared to be shorter in length. Additionally, several blank regions were identified, corresponding to sequences unique to E. stephanianum and lacking homology with other species. These results suggest inconsistent collinear block arrangements among the five mitochondrial genomes, indicating that the Es mitochondrial genome has undergone rearrangement compared to its relatives (Supplementary Table S6), more extensive results have been compiled in Supplementary Table S8.

**FIGURE 8:**
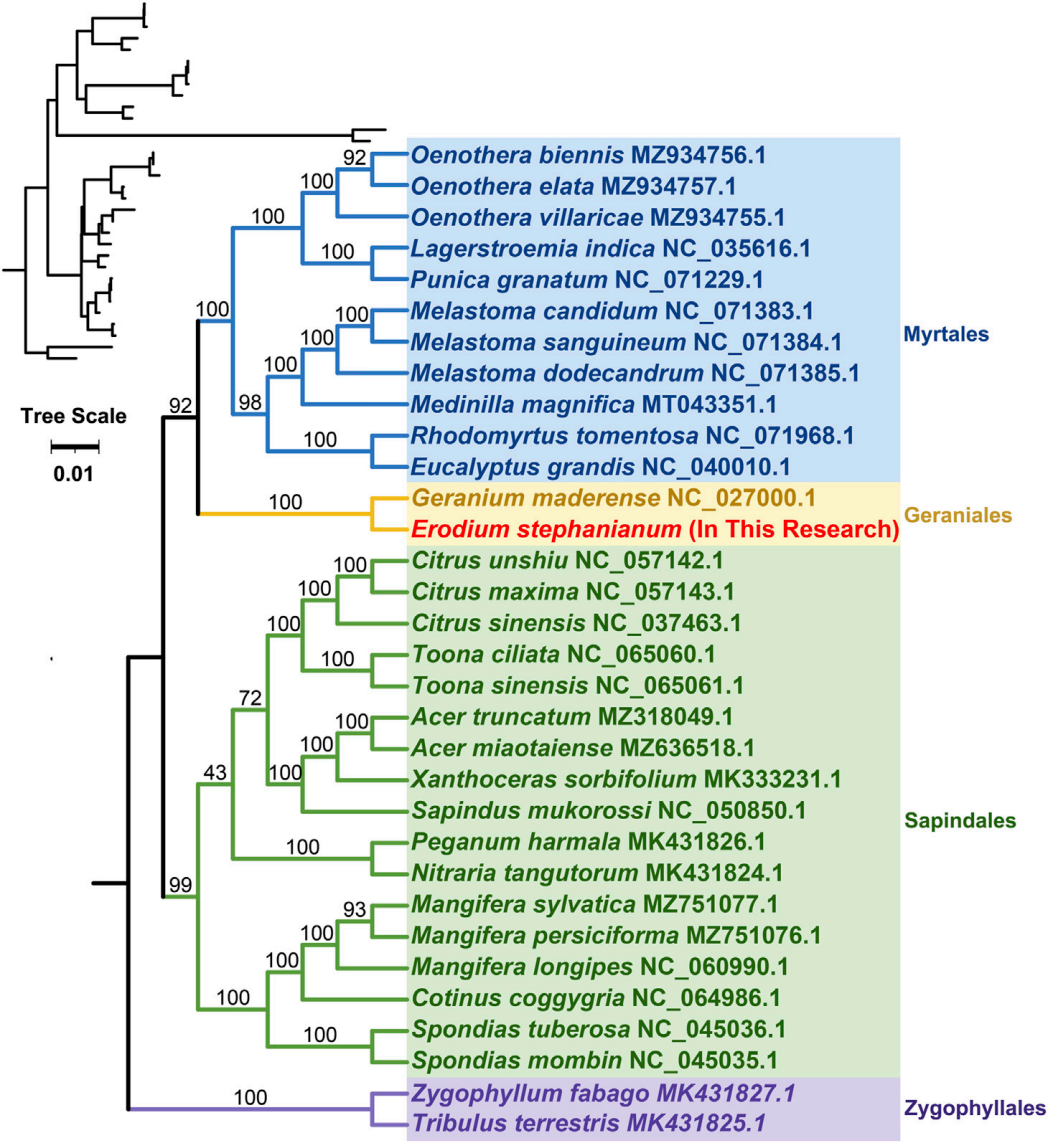
Phylogenetic tree of 32 angiosperms based on the sequences of 24 conserved mitochondrial PCGs. Two Zingiberaceae species were chosen as the outgroup. The number at each node is the bootstrap probability.

Phylogenetic trees were constructed for 32 species across four orders of angiosperms based on the DNA sequences of 24 conserved protein-coding genes (PCGs). The specific mitochondrial genome sequences of the studied plant species can be found in Appendix 1. The protein-coding genes examined included atp1, atp4, atp6, atp8, atp9, ccmB, ccmC, ccmFC, ccmFN, cob, cox1, cox2, cox3, matR, mttB, nad1, nad2, nad4, nad4L, nad5, nad6, nad9, and rps3. The mitochondrial genomes of two species from the family Zingiberaceae were designated as the outgroup (Supplementary Table S7). The phylogenetic topology based on mitochondrial DNA aligns with the most recent classification of the Angiosperm Phylogeny Group (APG). The species E. stephanianum belongs to the order Geraniales and the family Geraniaceae, clustering with the Geraniaceae species Geranium maderense, illustrating its close evolutionary relationship with Geranium maderense (NC_027000.1) (Figure 8).

### 3.6 The prediction of RNA editing

**FIGURE 9:**
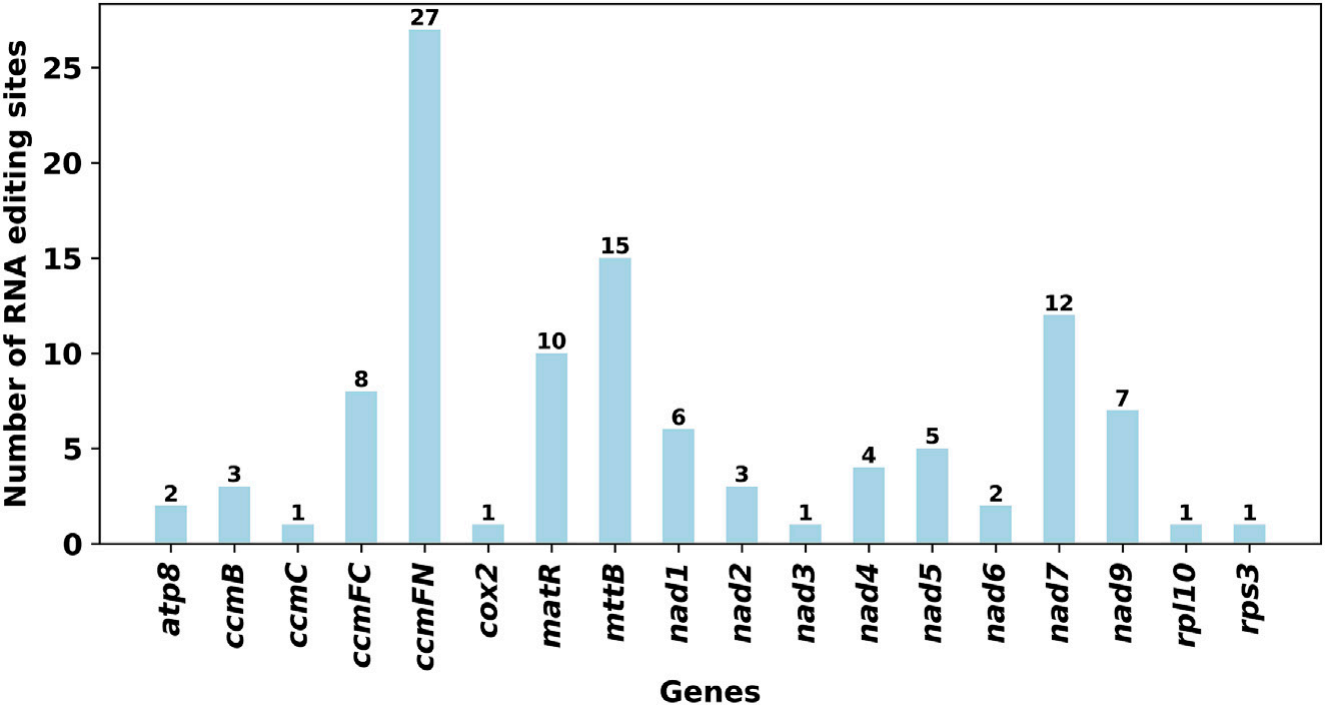
Characteristics of the RNA editing sites identified in PCGs of E. stephanianum mitogenome. Number of RNA editing sites predicted by individual PCGs using Deepred-mt. The abscissa shows the name of the gene, and the ordinate shows the number of edited sites.

RNA editing events were identified in 28 unique protein-coding genes (PCGs) from the mitochondrial genome of E. stephanianum using Deepred-mt. The cutoff value was set at 0.9. Under this threshold, a total of 109 potential RNA editing sites were detected across the 28 mitochondrial PCGs, all characterized by the conversion of cytosine (C) to uracil (U). The ccmFN gene exhibited the highest number of RNA editing sites, with 27 identified, followed by the mttB gene, which demonstrated 15 RNA editing events (Figure 9).

## 4 Discussion

The mitochondrial genome of Erodium stephanianum provides significant insights into the complexities of organelle evolution and interorganellar gene transfer. Our results demonstrate that the mitogenome exhibits a circular structure of 365,414 base pairs, comprising 28 unique protein-coding genes alongside important tRNA and rRNA genes. This genomic architecture is consistent with findings from other land plants, yet the presence of numerous plastid-derived sequences signifies a dynamic and ongoing process of gene transfer between the chloroplast and mitochondrial genomes (Wang et al., 2012; Liu et al., 2023; Ke et al., 2023).

The detection of 55 homologous fragments between the chloroplast and mitochondrial genomes, amounting to 58,305 base pairs, represents 15.96% of the total mitochondrial genome length. This gene transfer is particularly intriguing, as it mirrors observations in other plant species where such migrations have facilitated functional adaptations and evolutionary innovations. The existence of 33 complete genes-primarily protein-coding-and their successful integration into the mitochondrial genome underscores the evolutionary significance of these transfers. Notably, identified genes such as atpB, ndhA, and several others play critical roles in photosynthesis (Malinova et al., 2021; Lemaire et al., 1988; Li et al., 2022b) and energy metabolism (Lee, 2023; Rumeau et al., 2005), suggesting that adaptations may have occurred to enhance survival and fitness in varying ecological contexts (Hamilton et al., 2003; Huitric et al., 2010).

In addition to gene transfer, our study highlights the prevalence of repeated sequences within the E. stephanianum mitochondrial genome. The identification of 103 simple sequence repeats (SSRs) and numerous tandem repeats points to mechanisms of genetic stability and variability. Such repeats are critical for genome evolution, serving as markers for evolutionary change while also implicating potential roles in stress responses and adaptation processes. In previous analysis of the mitochondrial genome of Stemona sessilifolia, we identified a considerable number of simple sequence repeats (SSRs) and tandem repeats, specifically totaling 335 and 33, respectively. This finding aligns closely with the characteristics observed in E. stephanianum, which also exhibits a similar distribution of SSRs and tandem repeats. The presence of these repetitive elements suggests a potential evolutionary conservation of these genomic features across different species, possibly due to their role in genomic stability and adaptability (Xie et al., 2024). The analysis of codon usage further reveals a directional preference, particularly for specific amino acids, indicative of a structured evolutionary pathway that may enhance translational efficiency in mitochondrial gene expression (Varre et al., 2019; Bhattacharyya et al., 2002).

Phylogenetic analyses position E. stephanianum within the Geraniales order, closely related to Geranium maderense. The consistency of mitochondrial DNA phylogeny with the Angiosperm Phylogeny Group (APG) classification supports the integrity of our mitochondrial sequencing approach. It emphasizes the role of these organelle genomes in elucidating phylogenetic relationships across diverse angiosperm taxa. The resulting topology not only reinforces the evolutionary relationship but also serves as a template for future inquiries into the phylogenetic implications of mitochondrial evolution in relation to species diversification.

Moreover, our study uncovered a considerable number of RNA editing sites, predominantly characterized by C to U conversions. The high frequency of editing events in genes such as ccmFN (Sun et al., 2015; Nie et al., 2024) and mttB (Xu et al., 2024; Li et al., 2024) highlights the potential for post-transcriptional modifications to adapt mitochondrial functions in response to environmental perturbations. This phenomenon, prevalent in plant mitochondrial genomes (Zhang et al., 2021; Shavlakadze et al., 2023), may increase the robustness of gene expression under varying physiological conditions. The occurrence of RNA editing events in the ccmFN and mttB genes across wild barley (Ramadan et al., 2023), maize (Sun et al., 2015), and Fabaceae (Choi et al., 2020) highlights the evolutionary significance of post-transcriptional modifications in enhancing plant adaptability. These findings demonstrate that these editing events are not merely incidental but play crucial roles in vital physiological processes, particularly seed development and stress tolerance.

In conclusion, the complementation of sequencing technologies has illuminated the intricate dynamics of mitochondrial genomics in Erodium stephanianum. The gene transfer events identified provide compelling evidence of evolutionary adaptability, offering a foundation for further research into the mechanisms driving these changes. The implications of our findings extend beyond E. stephanianum, suggesting broader evolutionary trends in mitochondrial genome organization and function across diverse plant lineages.

## Data Availability

The datasets presented in this study can be found in online repositories. The names of the repository/repositories and accession number(s) can be found below: GenBank: https://www.ncbi.nlm.nih.gov/Genbank/, accession number PV575339.1; SRA: https://www.ncbi.nlm.nih.gov/sra/, accession number PRJNA1256539.

## Supplementary material

Supplementary Figure S1 Alternative conformation graph 1 of the mitochondrial genome of E. stephanianum.

Supplementary Figure S2 Alternative conformation graph 2 of the mitochondrial genome of E. stephanianum.
