# Supplementary figures and images for: Mitochondrial genome complexity in Erodium stephanianum (Geraniaceae): nanopore sequencing reveals chloroplast gene transfer and DNA rearrangements
Source: Front Genet. 2025 Jul 15;16:1641368. doi: 10.3389/fgene.2025.1641368 (PMC12303824; doi:10.3389/fgene.2025.1641368)

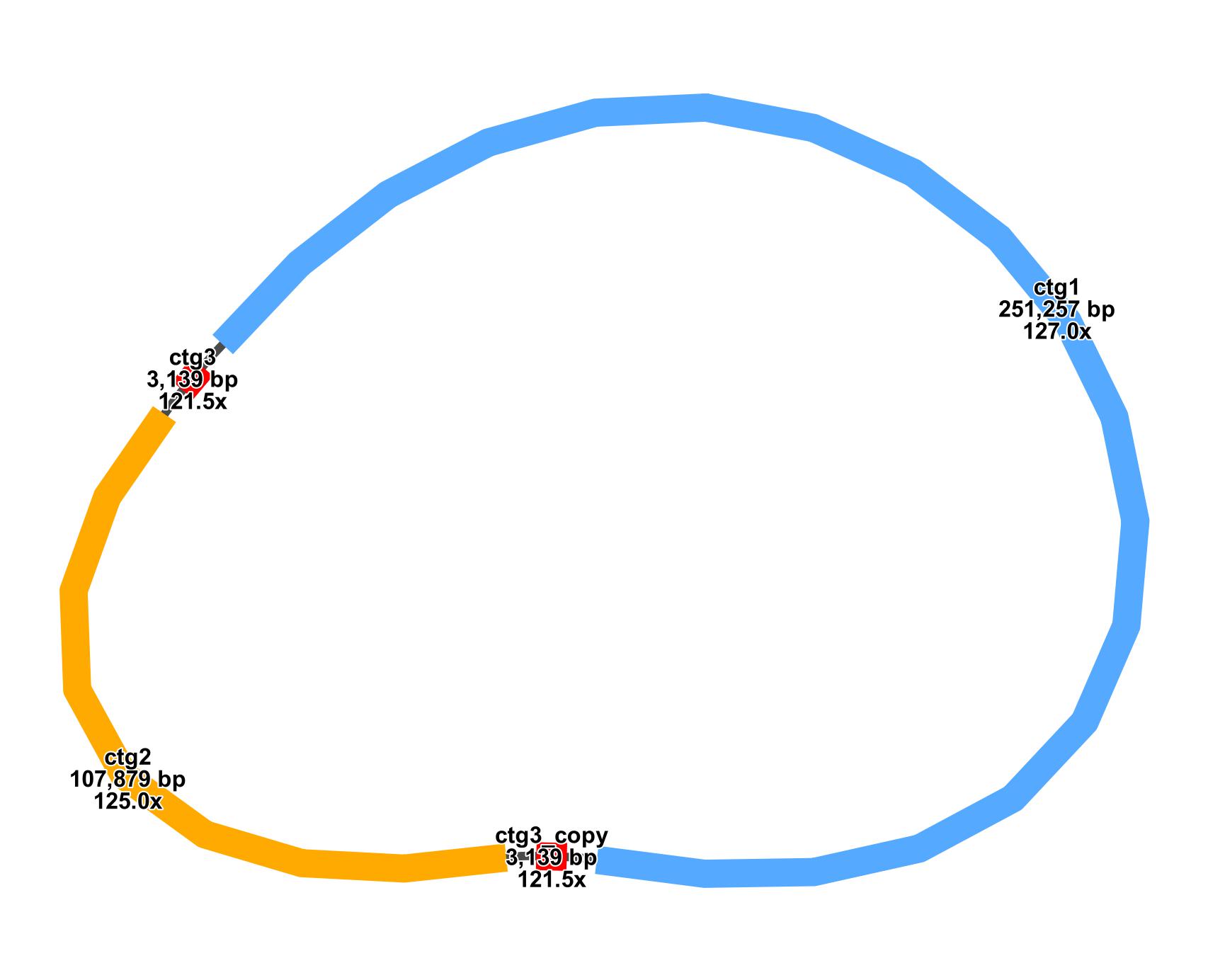

Supplement: Supplementary file 3 [file Image1.jpeg]

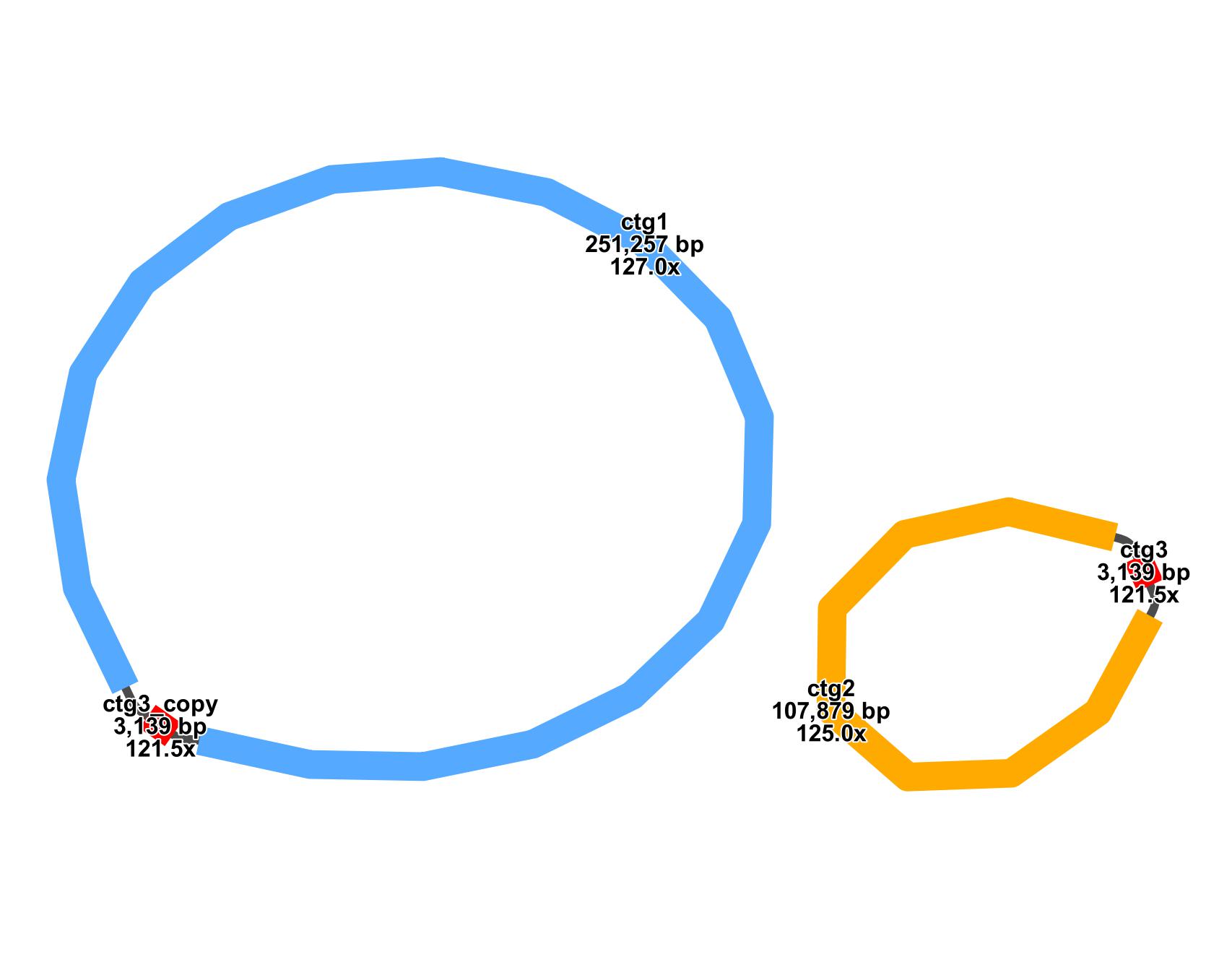

Supplement: Supplementary file 4 [file Image2.jpeg]
